# Animal Diet
*The Philosophy of the Stomach; or an Exclusively Animal Diet is the Most Wholesome and Fit for Man. By Bernard Moncriff. London: Longmans. 1856.


**Published:** 1856-10

**Authors:** 


### ANIMAL DIET.*

Mr. Bernard Moncriff's *Philosophy of the Stomach* is written to show that men could and should live exclusively on an animal diet. The author himself has thus subsisted for eighteen months. Fair readers of the *Journal of Public Health*, here is a good time come for one of you; for thus writes Mr. Moncriff. "I am yet unmarried, and entertain the natural wish to meet with a well educated lady, worthy of my sympathies, and reciprocating them, who should feel inclined"—ah! here's the rub—"to embrace my dietetical principles, or at least be so much in their favour as to allow" —Mr. Moncriff is evidently reckoning without his host at this point—"our children to be brought up in the same principles." Poor Mr. Moncriff! Ladies fair, grant him your smiles, as an overpowering recompense for the stern truth we are going to tell; viz., that this self-written hero has diluted a dozen lines of fact matter in ninety-two pages of egotistical nonsense and half-learned science.

* The Philosophy of the Stomach; or an Exclusively Animal Diet is the Most Wholesome and Fit for Man. By Bernard Moncriff. London: Longmans. 1856.

